# Separation of Membrane Vesicles and Cytosol from Yeast, Cultured Cells, and Bacteria in a Small Volume Self-Generated Gradient in a Fixed-Angle Rotor

**DOI:** 10.1100/tsw.2002.833

**Published:** 2002-06-12

**Authors:** John Graham

**Affiliations:** School of Biomolecular Sciences, Liverpool John Moores University, UK

**Keywords:** protein localization, cytosol, membrane vesicles, yeast, cultured cells, bacteria, OptiPrep™, iodixanol, discontinuous gradient, flotation, viscosity

## Abstract

There are many situations when it is necessary to separate rapidly and efficiently a cytosolic and a membrane vesicle fraction from yeast, cultured cells, or from bacteria. This Protocol Article describes the flotation of the vesicles through a self-generated gradient from a dense sample zone using the low-viscosity medium iodixanol. As the sample is exposed to the g_max_ the tendency of the proteins to sediment overcomes any diffusion in the opposite direction and are therefore completely separated from the vesicles.

**METHOD TYPE:** extraction, isolation, purification and separation

**SUB METHOD TYPE:** centrifugation

## INTRODUCTION

There are many situations where it is necessary to provide an effective and efficient separation of membrane vesicles from cytosolic proteins in order to determine whether a particular protein is either localized to a membrane or to the cytosol (or both). Ref. [1] describes a flotation method in a preformed discontinuous iodixanol gradient using a swinging-bucket rotor.

Ref. [2] describes a self-generated gradient system specifically for determining the location of a large protein complex; in this method a postnuclear supernatant is adjusted to 30% iodixanol and centrifuged for 1 h at 350,000*g*. Because the complex is rapidly sedimenting, it is completely resolvable from plasma membrane vesicles under these conditions. These conditions would not however be generally applicable to other smaller, less rapidly sedimenting cytosolic protein.

This Protocol Article describes a combination strategy in which the membrane vesicles float through a self-generated gradient from a dense load zone. It was developed by Du and Novick[3] for determining whether the GTPase activating protein Gyp1p was membrane-bound in yeast, but the method is more widely applicable to any protein in any cell type. It may be necessary follow the localization temporally and the method used by Du and Novick[3] is ideally suited to multiple samples because of simple tube filling using open-topped (1 ml) polycarbonate tubes for a fixed-angle rotor.

## MATERIALS (YEAST)

OptiPrep™ (60% w/v, iodixanol)
OptiPrep™ Diluent (OD): 2.4 M sorbitol, 6 mM EDTA, 120 mM tetraethylammonium acetate, pH 7.2
Working Solution (WS) of 50% (w/v) iodixanol: mix 5 vol of OptiPrep™ with 1 vol of OD
Lysis Buffer (LB): 0.4 M sorbitol, 1 mM EDTA, 20 mM tetraethyl-ammonium acetate, pH 7.2
Include protease inhibitors in solutions as required

## MATERIALS (MAMMALIAN CELLS)

OptiPrep™ (60% w/v, iodixanol)
OptiPrep™ Diluent (OD): 0.25 M sucrose, 6 mM EDTA, 120 mM Hepes-NaOH, pH 7.4
Working Solution (WS) of 50% (w/v) iodixanol: mix 5 vol of OptiPrep™ with 1 vol of Solution B
Homogenization Buffer (HB): 0.25 M sucrose, 1 mM EDTA, 20 mM Hepes-NaOH, pH 7.4
Include protease inhibitors in solutions as required. See Note 1 regarding other suitable solutions.

## EQUIPMENT

Microcentrifuge
Microultracentrifuge with small-volume fixed angle rotor: Beckman TLA120.2, TLA100.2, Sorvall S120-AT2, S150-AT or equivalent (see Notes 2 and 4)
Syringe and metal cannula for underlayering
Gradient unloader – Labconco Auto Densi-flow (optional) or automatic pipette

## METHOD

This procedure is adapted from Ref. [3].
Carry out all operations at 0–4°C.

1. Centrifuge the spheroplast lysate or cell homogenate at 1000*g* for 5 min to remove nuclei and unbroken cells.
2. Remove the supernatant and adjust to 40% iodixanol by mixing with WS (1 + 4 vol, respectively).

3. Prepare a solution of 35% iodixanol by diluting WS with LB or HB; place 0.9 ml in a tube for the ultracentrifuge fixed-angle rotor and underlayer it with the postnuclear supernatant in 40% iodixanol.
4. Transfer to a tube for the ultracentrifuge fixed-angle rotor and centrifuge at 120,000 rpm for 3 h (see Note 3).
5. Unload the gradient using an automatic pipette or a Labconco Auto Densi-flow fractionator in approx. 0.1 ml fraction (see Notes 4 and 5).

## ANALYSIS

Spectrophotometric (above 340 nm) analysis of enzymes, SDS-PAGE, and immunoprecipitation can be carried out in the presence of iodixanol. None of the common enzyme markers for membranes are inhibited by iodixanol[4]. If however, because the vesicles or proteins are not at a sufficiently high concentration for analysis, or if some particular functional inhibition is apparent, then the iodixanol can be easily removed. Vesicles suspensions should be diluted with 2 vol of HB, to reduce the density and viscosity of the suspension and after sedimentation at 100,000–150,000*g* for 45 min, the pellet can be suspended in an appropriately small volume of buffer. Removal of iodixanol from soluble proteins is best achieved by ultrafiltration through microcentrifuge cones, such as those in the Vectaspin® range manufactured by Whatman.

## NOTES

1. Use whatever homogenization solution is appropriate for the cells. Some mammalian cells require a hypo-osmotic solution for efficient cell rupture; if this is the case then adjust it to 0.25 M sucrose as soon as possible. If the HB contains low concentrations of other reagents such as DTT or MgOAc then these can be included in the OD at 6× the normal concentration so that they are present in the gradient at the same concentration as in the HB.
2. Almost any high-performance fixed-angle rotor can be used, as long as the sedimentation path length of the tube is less than 20 mm[5].
3. With such small sedimentation path length rotors it is very likely that the centrifugation time could be reduced to 2 h without seriously affecting the resolution of the gradient.
4. If the fractionation is carried in Beckman Optiseal™ tubes in a near-vertical rotor (e.g., Beckman TLN100), the volume of sample and gradient will need increasing (the tubes hold approx. 3.1 ml) but the collection of the gradient is more flexible – tube puncture can be used. Because of the short sedimentation path length of this rotor, the centrifugation time will not need to be increased. For more information on gradient harvesting see Ref. [6].
5. The vesicles band in the top half of the gradient, while all soluble proteins are confined to the bottom of the gradient (see [3]).

## ACKNOWLEDGEMENTS

The author and TheScientificWorld wish to thank Axis-Shield PoC, AS, Oslo, Norway for their kind permission to adapt OptiPrep™ Application Sheet S31 in the preparation of this Protocol Article
